# Jun Is Required in *Isl1*-Expressing Progenitor Cells for Cardiovascular Development

**DOI:** 10.1371/journal.pone.0057032

**Published:** 2013-02-21

**Authors:** Tao Zhang, Junchen Liu, Jue Zhang, Eldhose B. Thekkethottiyil, Timothy L. Macatee, Fraz A. Ismat, Fen Wang, Jason Z. Stoller

**Affiliations:** 1 Division of Neonatology, Department of Pediatrics, Perelman School of Medicine at the University of Pennsylvania, Children’s Hospital of Philadelphia, Philadelphia, Pennsylvania, United States of America; 2 Center for Cancer and Stem Cell Biology, Institute of Biosciences and Technology, Texas A & M Health Science Center, Houston, Texas, United States of America; 3 Morgridge Institute for Research, Madison, Wisconsin, United States of America; 4 Department of Pathology and Laboratory Medicine, Perelman School of Medicine at the University of Pennsylvania, Philadelphia, Pennsylvania, United States of America; 5 Division of Cardiology, Department of Pediatrics, Perelman School of Medicine at the University of Pennsylvania, Children’s Hospital of Philadelphia, Philadelphia, Pennsylvania, United States of America; Centro Cardiologico Monzino, Italy

## Abstract

Jun is a highly conserved member of the multimeric activator protein 1 transcription factor complex and plays an important role in human cancer where it is known to be critical for proliferation, cell cycle regulation, differentiation, and cell death. All of these biological functions are also crucial for embryonic development. Although all *Jun* null mouse embryos die at mid-gestation with persistent truncus arteriosus, a severe cardiac outflow tract defect also seen in human congenital heart disease, the developmental mechanisms are poorly understood. Here we show that murine Jun is expressed in a restricted pattern in several cell populations important for cardiovascular development, including the second heart field, pharyngeal endoderm, outflow tract and atrioventricular endocardial cushions and post-migratory neural crest derivatives. Several genes, including *Isl1*, molecularly mark the second heart field. *Isl1* lineages include myocardium, smooth muscle, neural crest, endocardium, and endothelium. We demonstrate that conditional knockout mouse embryos lacking Jun in *Isl1*-expressing progenitors display ventricular septal defects, double outlet right ventricle, semilunar valve hyperplasia and aortic arch artery patterning defects. In contrast, we show that conditional deletion of Jun in *Tie2-*expressing endothelial and endocardial precursors does not result in aortic arch artery patterning defects or embryonic death, but does result in ventricular septal defects and a low incidence of semilunar valve defects, atrioventricular valve defects and double outlet right ventricle. Our results demonstrate that Jun is required in *Isl1*-expressing progenitors and, to a lesser extent, in endothelial cells and endothelial-derived endocardium for cardiovascular development but is dispensable in both cell types for embryonic survival. These data provide a cellular framework for understanding the role of Jun in the pathogenesis of congenital heart disease.

## Introduction

Jun (a.k.a. c-Jun) is a highly conserved member of the multimeric activator protein 1 (AP-1) transcription factor complex [1]. The AP-1 protein complexes are a heterogeneous group of transcriptionally active dimers and include members of the Jun family (Jun, Jund, Junb), Fos family (c-Fos, Fosb, Fosl1, Fosl2) and other transcription factor families such as ATF and Maf [2]. Several *Jun* mutant mice have been generated to study AP-1 function. While *Jun* heterozygous mice are normal [3], all *Jun* null embryos die between E12.5 and E14.5 with persistent truncus arteriosus (PTA) [3], [4], [5]. PTA is a severe developmental cardiac abnormality seen in many patients as an isolated finding or as part of a syndrome such as DiGeorge/22q11 deletion syndrome. Jun proteins can form homo- or heterodimers to differentially regulate transcription [1]. Examination of the promiscuity of these dimer protein-protein interactions has revealed that as part of a DNA-binding complex, Jun is critical for multiple biological processes including cell proliferation, apoptosis, cell cycle progression and differentiation [6], [7], [8], [9]. Although these cellular phenomena are critical for mammalian development and for diseases such as cancer, data regarding the role of Jun during embryogenesis is limited.

The cardiac outflow tract (OFT) incorporates the lineages of multiple cardiac progenitors and its development is dependent upon the complex interaction of several cell types. Neural crest (NC) cells migrate from the dorsal neural tube to the developing aorticopulmonary septation complex to mediate septation of the truncus arteriosus into the main pulmonary artery and aorta [10]. These NC cells contribute to the OFT endocardial cushion mesenchyme which is also comprised of endothelial-derived endocardial cells [11]. Second heart field (SHF) progenitors contribute to the OFT myocardium and smooth muscle [12], [13] while endothelial progenitors give rise to the mature endothelial cells and semilunar valves of the OFT [14], [15]. Defects seen in *Jun* null embryos are striking and may be mediated by Jun function in one or more of these cell populations involved in OFT development. Here we show that murine Jun is expressed in a restricted pattern in several cell populations important for cardiovascular development, including the SHF, pharyngeal endoderm, OFT endocardial cushions, atrioventricular (AV) endocardial cushions and post-migratory NC derivatives. Using tissue-specific conditional deletion studies in mice, we demonstrate that Jun is required in *Isl1*-expressing progenitors and, to a lesser extent, in endothelial cells and endothelial-derived endocardium for cardiovascular development but is dispensable in both cell types for embryonic survival.

## Results

### Jun is Detected during Mid-gestation in Restricted Cell Populations

**Figure 1:**
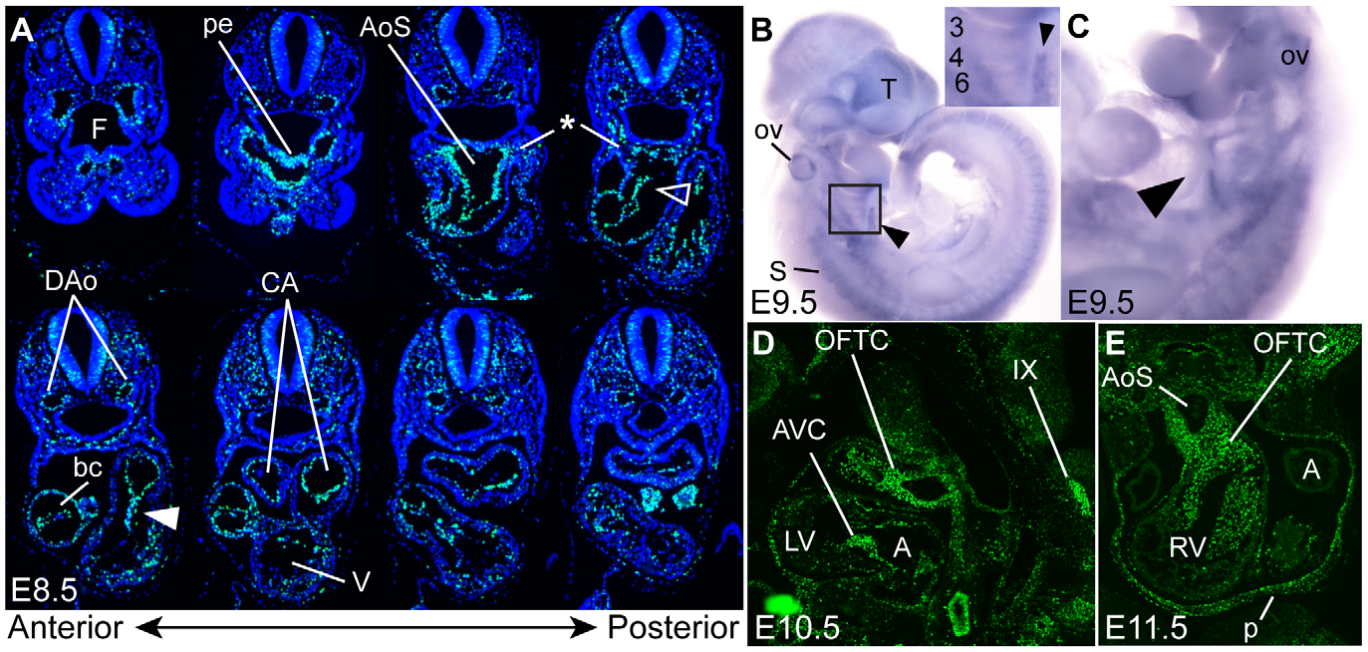
Jun is expressed in cell populations known to contribute to cardiogenesis. (**A**) Transverse sections along the full anteroposterior axis showing Jun immunostaining (green) in the pharyngeal endoderm (pe), SHF mesoderm (*), OFT cushions (open arrowhead), AV cushions (closed arrowhead), dorsal aortae (DAo) and common atrial chamber (CA). (**B, C**) Whole mount *in situ* hybridization of *Jun* expression in the SHF (arrowhead), somites (S), otic vesicle (ov) and telencephalon (T). Inset is a higher power image of the area shown in the black box in panel B showing *Jun* expression in aortic arch arteries 3, 4 and 6. Parasagittal (**D**) and transverse (**E**) sections showing Jun expression in endocardial cushions, and cranial nerve IX. A, atrium; AoS, aortic sac; AVC, atrioventricular cushion; bc, bulbus cordis; F, foregut; LV, left ventricle; OFTC, outflow tract cushion p, pericardium; RV, right ventricle; V, ventricle.

Several cell populations are important for normal OFT development and septation. The 100% incidence of PTA in *Jun* null embryos indicates that Jun is clearly required in one or more of these cell populations. An overview of *Jun’s* spatial and temporal expression pattern during embryonic development in the mouse is lacking in the literature, particularly prior to E14.5. In limited expression analyses by *in situ* hybridization and Northern blot, it has been reported that *Jun* mRNA is expressed in the developing heart, cartilage, gut, central nervous system, lung, kidney, adrenal gland and placenta of the developing mouse [16], [17], [18], [19], [20]. To determine the specific cell populations in which Jun might be functioning to regulate cardiac morphogenesis, we examined the expression of *Jun* by *in situ* hybridization and immunohistochemistry at several stages of embryonic development between E8.5 and E15.5. Our Jun expression analysis revealed expression in multiple tissues important for heart development and aortic arch artery remodeling. At E8.5, Jun was expressed in the pharyngeal endoderm, dorsal aortae, common atrial chamber, endocardial cushions and in regions populated by SHF mesoderm (Fig. 1A). The anterior SHF expression was stronger than the posterior SHF (Fig. 1A). The expression of *Jun* in the SHF was also evident at E9.5 by whole mount *in situ* hybridization (Fig. 1B, C). This is consistent with our previous observation of Jun expression in SHF-derived OFT myocardium [21]. At E9.5, *Jun* was expressed in the otic vesicle, telencephalon, somites, and aortic arch arteries (Fig. 1B, C). The expression in the telencephalon, somites and pharyngeal arches is consistent with publically available *in situ* hybridization data at E11 (http://goo.gl/DoJro) [22]. At E10.5, Jun was highly expressed in the OFT endocardial cushions, AV endocardial cushions and cranial nerve IX (Fig. 1D). The high levels of Jun expression in the OFT endocardial cushions persists until E11.5 (Fig. 1E), where expression in pericardium (Fig. 1E) and dorsal root ganglia (data not shown) was also evident. At E15.5, Jun was broadly expressed in the myocardium and both the semilunar and AV valves (Fig. S1).

### Conditional Deletion of *Jun* in *Isl1*-expressing Progenitors Results in Severe Cardiovascular Malformations

**Figure 2:**
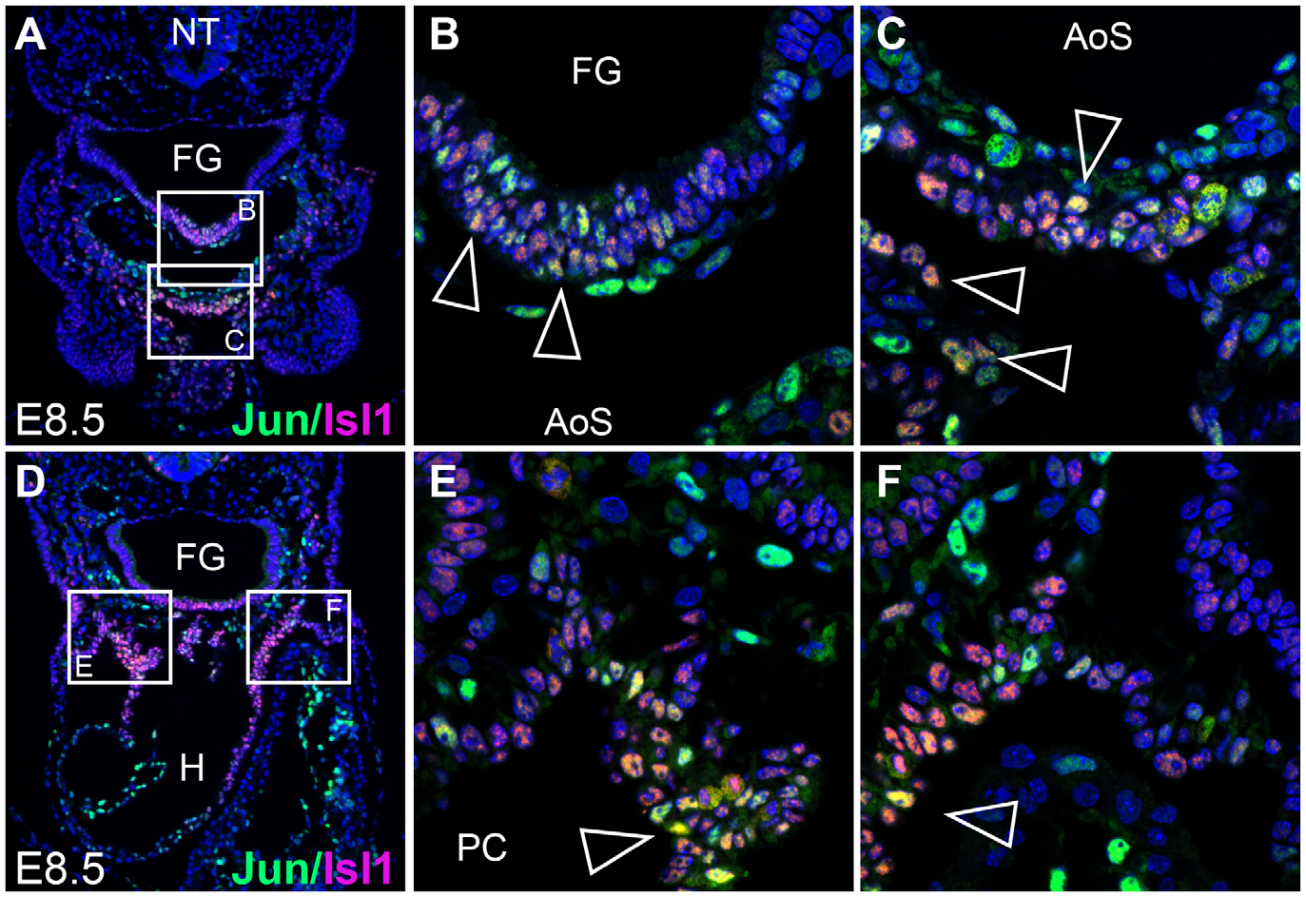
Jun and Isl1 are co-expressed in a subset of cells in the developing outflow tract. (**A, D**) Transverse sections of an E8.5 wild-type embryo showing Jun (green) and Isl1 (pink) immunostaining in anterior SHF mesoderm and pharyngeal endoderm. Sections were co-stained with DAPI to illustrate nuclei. (**B, C, E, F**) Higher power confocal images of the areas indicated by white boxes in panels A and D. Cell populations co-expressing Jun and Isl1 are indicated by the open arrowheads. AoS, aortic sac; FG, foregut; H, heart; NT, neural tube; PC, pericardial cavity.

*Jun* is expressed in the SHF (Fig. 1) and SHF-derived myocardium (Fig. S1) [21]. The SHF comprises a specialized subset of cardiac progenitor cells derived from early splanchnic mesoderm [13]. These progenitors are marked by the expression of genes such as *Isl1* [13], [23], [24] and play a critical role in the development of the right ventricle and OFT. To determine if Jun is expressed in *Isl1*-positive cells, we examined the expression of Jun and Isl1 at E8.5 using immunohistochemistry. Our analysis using confocal microscopy revealed co-localization of Jun and Isl1 in the anterior SHF mesoderm (Fig. 2A, C–F) and pharyngeal endoderm (Fig. 2A, B).

**Figure 3:**
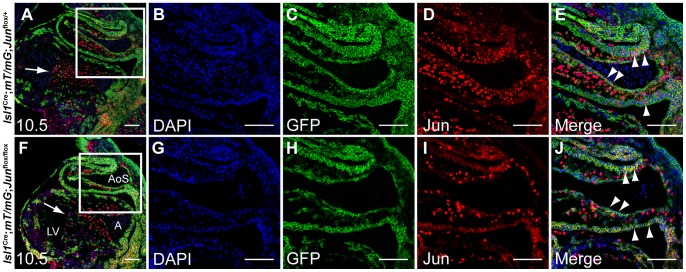
Jun is efficiently deleted in derivatives of *Isl1*-expressing cells in the outflow tract. Saggital sections of E10.5 *Isl1* ^Cre/+^ *; mT/mG; Jun* ^flox/+^ (**A**) and *Isl1* ^Cre/+^ *; mT/mG; Jun* ^flox/flox^ (**F**) embryos co-immunostained with anti-GFP (green) and anti-Jun (red) and analyzed by confocal microscopy showing nuclear Jun expression. *Isl1* ^Cre/+^-expressing progenitors were marked with GFP expression using the mT/mG double-fluorescent Cre reporter mice. Sections were co-stained with DAPI to illustrate nuclei. (**B–E, G–J**) Higher power images of the areas indicated by white boxes in panels A, F. Jun is efficiently deleted in derivatives of *Isl1*-expressing progenitors located in the OFT (arrowheads) whereas AV cushion cells (arrows), not derived from *Isl1*-expressing progenitors, express Jun in both control (**E**) and mutant embryos (**J**). A, atrium; AoS, aortic sac; LV, left ventricle. Scale bar: 100 µm.

To determine if Jun is required in *Isl1*-expressing progenitors, we performed a conditional deletion of *Jun* using *Isl1* ^Cre/+^ knock-in mice [25], [26]. There are several mutant mice expressing Cre recombinase in *Isl1*–expressing lineages [25], [26], [27], [28]. To avoid the variable Cre activity observed with the *Isl1*-IRES-Cre mice (*Isl1^tm1(cre)Tmj^*) [25], [27], we utilized the *Isl1* ^Cre/+^ knock-in mice [25] which have been extensively characterized and shown to drive Cre recombinase expression as early as E8-8.5 [25], [26]. *Jun* conditional knockout mice (*Jun* ^flox/flox^) [7] have been validated through tissue-specific deletion in liver [7], [29], neuroepithelial cells [30], keratinocytes [31], [32] and notochord and sclerotome [33]. To determine if Jun was deleted in the *Isl1* lineage, we performed Jun immunostaining of E10.5 *Isl1* ^Cre/+^; *Jun* ^flox/flox^; *mT/mG* and control embryos. Cre-mediated recombination in mT/mG embryos [34] indelibly marks *Isl1*-derived progenitors with GFP expression. Confocal microscopy of *Isl1* ^Cre/+^; *Jun* ^flox/flox^ and control embryonic sections revealed that Jun was efficiently deleted in derivatives of *Isl1*-expressing progenitors residing in the OFT (Fig. 3).

**Figure 4:**
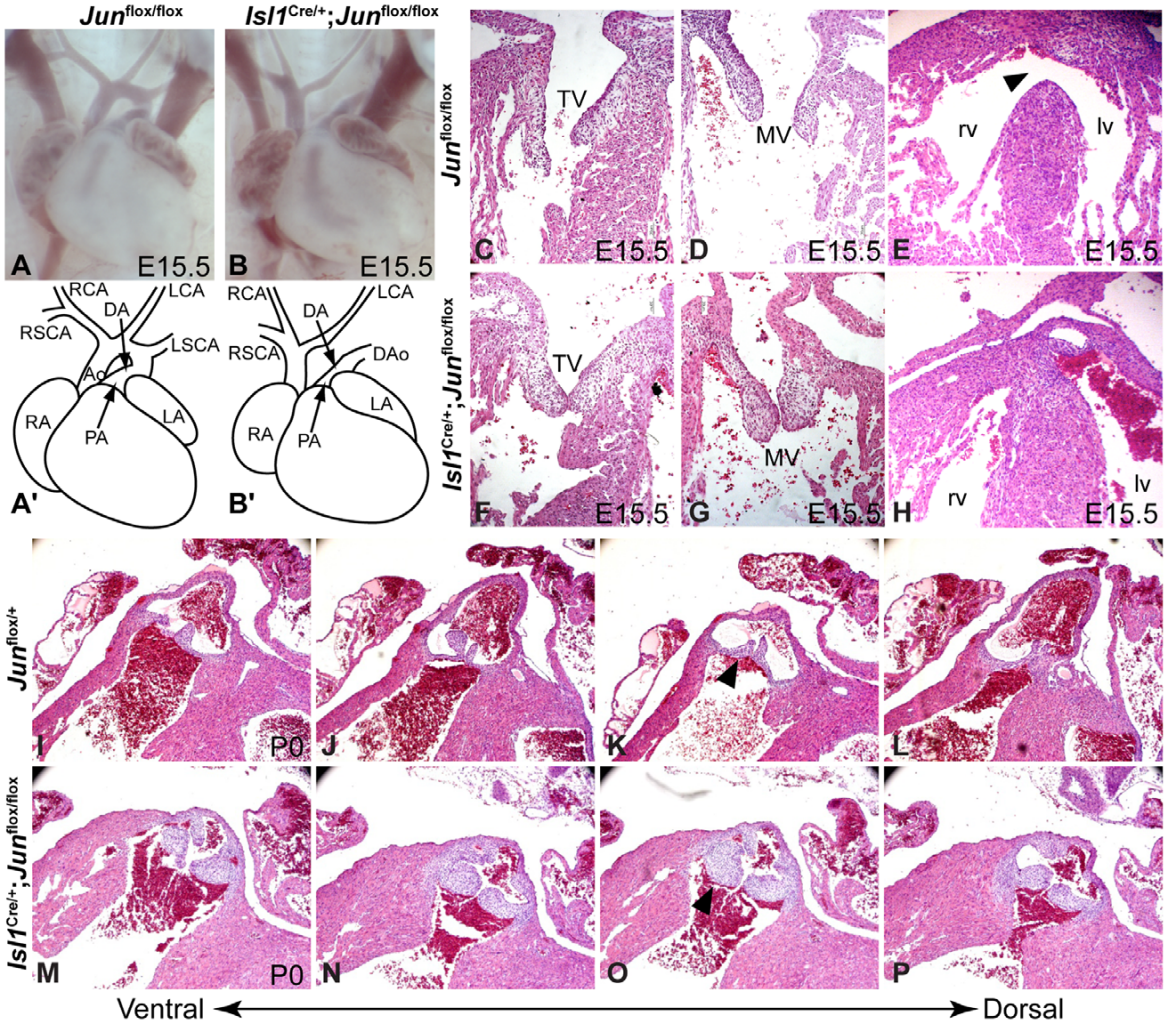
*Isl1*-specific deletion of *Jun* results in cardiovascular defects. Compared to control embryos (**A, A', C–E, I–L**), the loss of *Jun* in *Isl1*-expressing precursors results in IAA (**B, B'**), VSD (arrowhead, **E**), and enlarged and hyperplastic pulmonary valve leaflets (arrowhead, **O**). The atrioventricular valves are unaffected (**C, D, F, G**). Ao, aorta; CA, carotid artery; DA, ductus arteriosus; DAo, descending aorta; LA, left atrium; lv, left ventricle; MV, mitral valve; PA, pulmonary artery; RA, right atrium; rv, right ventricle; SCA, subclavian artery; TV, tricuspid valve.

*Isl1* ^Cre/+^ *; Jun* ^flox/flox^ embryos were present at the expected Mendelian ratios (E14.5-P0, n = 23, 100% of predicted; Table 1) and thus survived longer than *Jun* null embryos [3], [4], [5]. This suggests that Jun function in *Isl1*-expressing cells is not responsible for the embryonic mortality seen in *Jun* null embryos and supports the hypothesis of Hilberg and Eferl et al. that the embryonic death in *Jun* nulls is attributable to impaired hepatogenesis [3], [5] rather to the cardiac defects. We examined E14.5 to P0 *Isl1* ^Cre/+^ *; Jun* ^flox/flox^ and control embryos for cardiac defects. Upon careful examination, we found that 32% of *Isl1* ^Cre/+^ *; Jun* ^flox/flox^ conditional mutant embryos (n = 22) showed aortic arch artery remodeling defects (Fig. 4B, B′; Table 2). These defects included interrupted aortic arch (IAA) type B, hypoplasia of the B segment of the aortic arch and aberrant retro-esophageal right subclavian artery. OFT defects were observed in 88% of conditional mutant embryos (n = 8) and included ventricular septal defect (VSD), double outlet right ventricle, and semilunar valve hyperplasia (Fig. 4F, K–N; Table 2). Although there is some evidence that Isl1 progenitors may contribute to the mature AV valves [35], we observed very few GFP-expressing cells derived from *Isl1*-expressing progenitors cells in the developing AV cushions (Fig. 3A, F). Consistent with this observation, we did not observe any AV valve defects in *Isl1* ^Cre/+^ *; Jun* ^flox/flox^ embryos (Fig. 4G, H; Table 2).

**Table 1:** Summary of genotypes for *Jun* conditional mutants.

Genotype(E14.5-P0)	n (%)	Genotype (E15.5-P0)	n (%)
*Isl1* ^Cre/+^; *Jun* ^flox/+^	23 (25%)	*Tie2-*Cre; *Jun* ^flox/+^	12 (24%)
*Isl1* ^Cre/+^; *Jun* ^flox/flox^	23 (25%)	*Tie2-*Cre; *Jun* ^flox/flox^	10 (20%)
*Jun* ^flox/+^	26 (28%)	*Jun* ^flox/+^	15 (31%)
*Jun* ^flox/flox^	20 (22%)	*Jun* ^flox/flox^	12 (24%)
χ^ 2^	p = 0.94	χ^ 2^	p = 0.92

**Table 2:** Cardiovascular abnormalities in late gestation *Jun* conditional mutants.

E14.5–P0	*Jun* ^flox/+^ or *Jun* ^flox/flox^	*Isl1* ^Cre/+^ *; Jun* ^flox/flox^	*Tie2-Cre; Jun* ^flox/flox^
Aortic arch artery remodeling defects	0% (0/46)	32% (7/22)	0% (0/10)
Ventricular septal defect	0% (0/9)	88% (7/8)	43% (3/7)
Double outlet right ventricle	0% (0/9)	88% (7/8)	14% (1/7)
Mitral valve defect	0% (0/9)	0% (0/8)	14% (1/7)
Tricuspid valve defect	0% (0/9)	0% (0/8)	0% (0/7)
Pulmonary valve defect	0% (0/9)	88% (7/8)	14% (1/7)
Aortic valve defect	0% (0/9)	75% (6/8)	0% (0/7)

Because *Isl1* ^Cre/+^ is a loss-of-function allele [25], we looked for evidence of a genetic interaction between *Isl1* and *Jun*. Neither *Isl1* ^+/−^ nor *Isl1* ^Cre/+^ mice have discernible cardiac defects [23], [25], [36], thus heterozygosity of *Isl1* from the Cre knock-in could not account for the phenotype in *Isl1* ^Cre/+^ *; Jun* ^flox/flox^ mutants. We observed a single *Isl1* ^Cre/+^ *; Jun* ^flox/+^ embryo (1 of 22) with IAA type B. No other OFT or aortic arch abnormalities were noted among *Isl1* ^Cre/+^ *; Jun* ^flox/+^ embryos. While this suggests a subtle genetic interaction, it does not account for the significantly higher incidence of defects observed in *Isl1* ^Cre/+^ *; Jun* ^flox/flox^ embryos.

**Figure 5:**
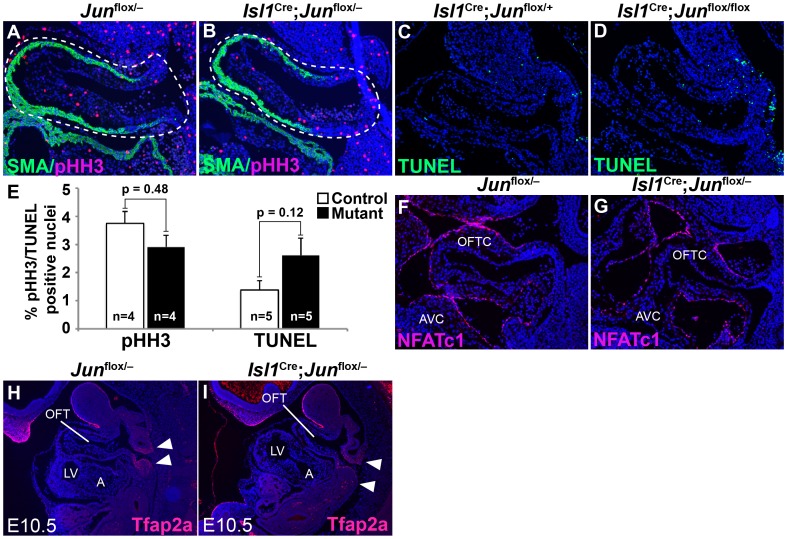
Effect of the loss of Jun in *Isl1*-expressing progenitors on proliferation, apoptosis, cardiac neural crest cells and the endocardium. Sagittal sections of E10.5 mutant (**B, D, G**) and control (**A, C, F**) embryos analyzed by pHH3 and NFATc1 immunostaining and by TUNEL assay. pHH3 immunostaining (pink) reveals similar proliferation rates in mutant and control OFT cells (**A, B**). Dotted lines show representative areas used for cell counting with ImageJ software. TUNEL assay reveals similar numbers of TUNEL-positive (green) OFT cells in mutant and control embryos (**C, D**). (**E**) Quantitative analysis of the percentage of pHH3- and TUNEL-positive OFT cells in serial sections showing a statistically insignificant trend toward less proliferation and more apoptosis in conditionally deleted embryos at E10.5. Results are expressed as mean ± SEM of the percent positive nuclei. The statistical significance of differences between groups was analyzed by the Student’s t-test. (**F, G**) Expression of the endocardial marker NFATc1 (pink), is not significantly different between conditional mutant and control embryos. Saggital sections of anti-Tfap2a immunostained *Jun* ^flox/−^ (**H**) and *Isl1* ^Cre/+^ *; Jun* ^flox/−^ (**I**) embryos showing no significant difference in Tfap2a-expressing neural crest cells (arrowheads) at E10.5. Sections were co-stained with DAPI to illustrate nuclei. A, atrium; AVC, atrioventricular cushion; LV, left ventricle; OFT, outflow tract; OFTC, outflow tract cushion; SMA, smooth muscle actin.

Given Jun’s role in proliferation and apoptosis [6], [7], we investigated whether an alteration in one or both of these cellular processes may contribute to the OFT defects observed in *Jun* mutant mice. Using phospho-histone H3 (pHH3) and TUNEL as markers of cell proliferation and apoptosis, we could not detect any statistically significant differences in the percent of pHH3- or TUNEL-positive cells in serial sections of the OFT of E10.5 *Isl1* ^Cre/+^ *; Jun* ^flox/flox^, *Isl1* ^Cre/+^ *; Jun* ^flox/−^ and control littermates (Fig. 5A–E). Although the differences were not statistically significant, there was a trend toward a decrease in proliferating cells and an increase in apoptosis in the OFT of mutant embryos. These findings suggest that the observed OFT defects may not be solely the result of altered proliferation or apoptosis, but raises the possibility that in combination, these alterations could contribute to the phenotype.

Endothelial progenitors, contributing to the developing OFT endocardial cushion mesenchyme, and cardiac NC cells play an important role in the development of the OFT [10], [11], [14], [15]. We used NFATc1 as an endocardial marker [14], [37] and Tfap2a (a.k.a. AP-2α) as a marker of migrating NC cells (and ectoderm) [36], [38] to determine if conditional deletion of Jun in *Isl1*-expressing progenitors affected the developing endocardium or NC cells. We could not detect any significant differences in NFATc1 or Tfap2a expression in E10.5 *Isl1* ^Cre/+^ *; Jun* ^flox/−^ embryos compared with control littermates (Fig. 5F–I). These findings suggest that the defects observed in *Isl1* conditional *Jun* mutant embryos are not attributable to a failure of endocardial formation or cardiac NC migration.

### Jun is Required in the Endocardium and Endothelial Progenitors for Valve and Ventricular Septum Formation but not for Aortic Arch Artery Remodeling

**Figure 6:**
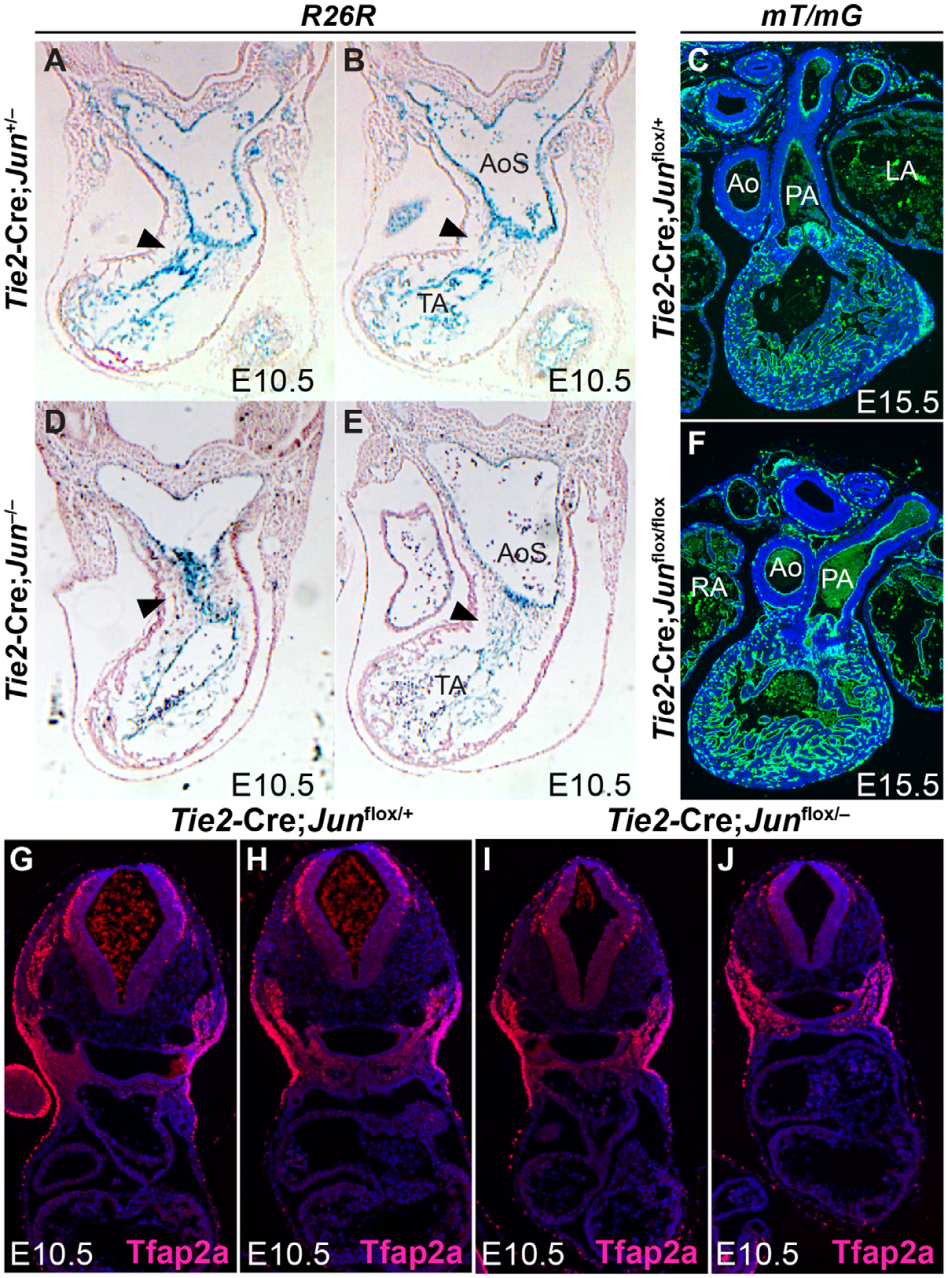
Loss of *Jun* does not alter the fate of *Tie2*-expressing endothelial derivatives or cardiac neural crest cells in the developing OFT. Transverse sections of X-gal stained *Jun* ^+/−^ *; Tie2-Cre;R26R* (**A, B**) and *Jun* ^−/−^ *; Tie2-Cre; R26R* (**D, E**) embryos showing no significant difference in endothelial derivatives (blue) populating the OFT endocardial cushion mesenchyme (arrowheads) at E10.5. Transverse sections of anti-GFP immunostained *Tie2-Cre; Jun* ^flox/+^ *; mT/mG* (**C**) and *Tie2-Cre; Jun* ^flox/flox^ *; mT/mG* (**F**) embryos showing no significant difference in endothelial derivatives (green) populating the semilunar valves, heart and blood vessels at E15.5. Transverse sections of anti-Tfap2a immunostained *Tie2-Cre; Jun* ^flox/+^ (**G, H**) and *Tie2-Cre; Jun* ^flox/−^ (**I, J**) embryos showing no significant difference in Tfap2a-expressing neural crest cells at E10.5. Sections were co-stained with DAPI to illustrate nuclei. Ao, aorta; AoS, aortic sac; LA, left atrium; PA, pulmonary artery; RA, right atrium; TA, truncus arteriosus.

Although *Isl1*-expressing progenitors populate the SHF mesoderm, *Isl1* lineages also include other cell populations such as the endocardium and scattered endothelial cells in the aortic arch arteries [13], [23], [24], [26], [39]. Endothelial cells, giving rise to the endocardium, undergo an epithelial-to-mesenchymal transformation to contribute to the OFT cushion mesenchymal tissue during OFT septation [11]. The endocardial cushions subsequently give rise to the valves of the mature heart [14], [15]. Our finding that Jun was strongly expressed in the OFT endocardial cushion mesenchyme (Fig. 1D, E) together with the prominent endocardial cushions noted in *Jun* null embryos [5] suggests that Jun may be required in the endothelial progenitors giving rise to endocardial cushion mesenchyme. Hence, we performed fate-mapping studies of endothelial cells in *Jun* mutant embryos. Endothelial cells and endothelial-derived endocardium were marked with ß-galactosidase or GFP expression by crossing transgenic *Tie2-Cre* mice with R26R or mT/mG Cre reporter mice [34], [40], [41]. *Tie2-Cre* mice have been extensively characterized and shown to drive expression specifically in the embryonic endothelium and endocardium as early as E7.5 [41]. In global *Jun* nulls at E10.5 (Fig. 6D, E), and in endothelial-specific conditional mutants at E15.5 (Fig. 6F), we observed the contribution of *Tie2*-expressing endothelial progenitors to the endothelial cushion mesenchyme and endothelial lining of the truncus arteriosus and aortic sac and to the semilunar valves in a pattern similar to control embryos (Fig. 6A–C). Although these fate mapping experiments suggest that Jun is not required in *Tie2*-expressing endothelial progenitors, it remains possible that the loss of Jun in the endocardium results in a functional defect resulting in cardiac defects or aortic arch artery remodeling defects.

**Figure 7:**
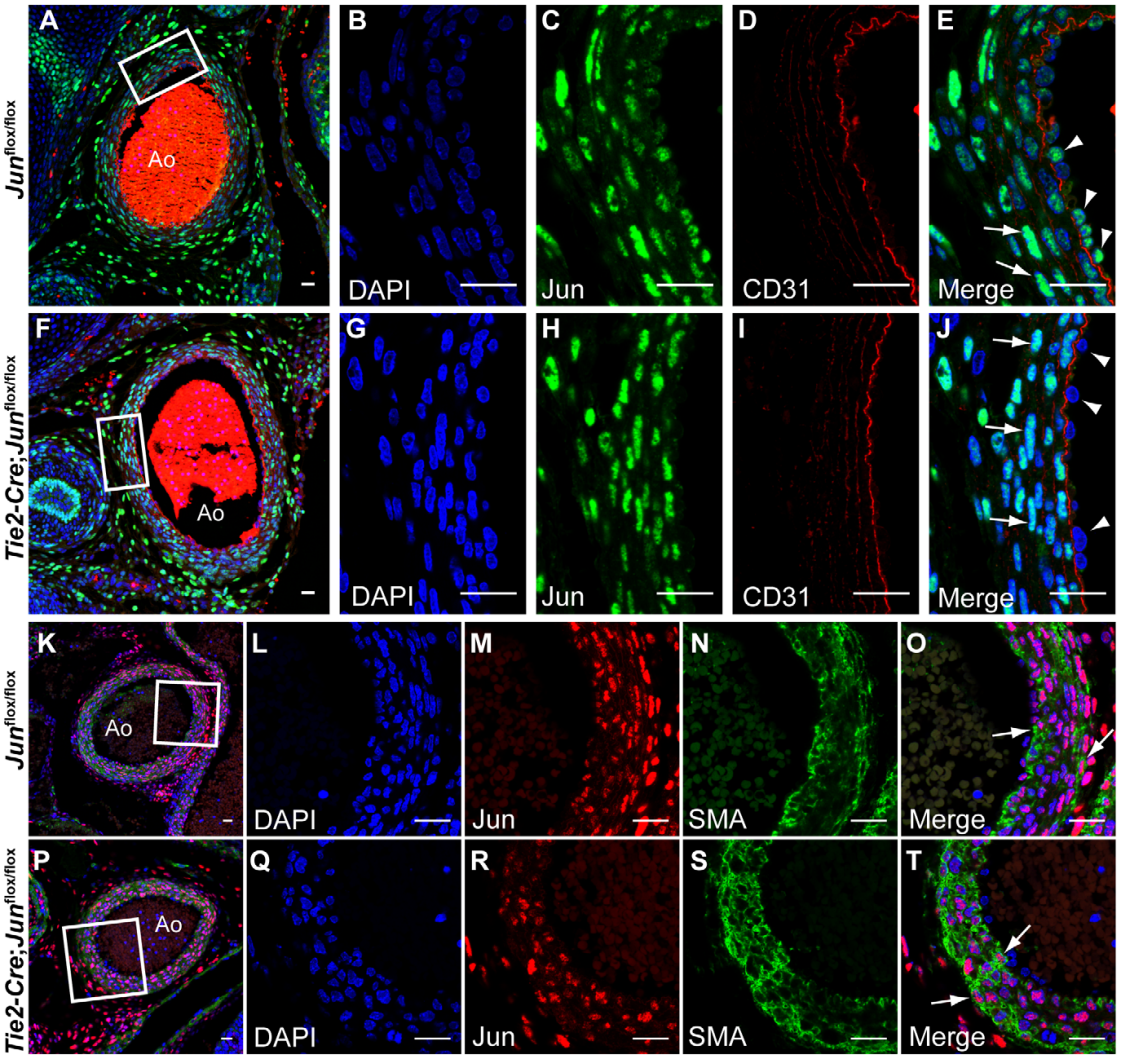
Jun is efficiently deleted in *Tie2*-expressing endothelial derivatives. Cross sections of E15.5 *Jun* ^flox/flox^ (**A, K**) and *Tie2-Cre; Jun* ^flox/flox^ (**F, P**) embryos co-immunostained with anti-CD31, anti-SMA and anti-Jun analyzed by confocal microscopy. Sections were co-stained with DAPI to illustrate nuclei. (**B–E, G–J**) Higher power images of the areas indicated by white boxes in panels A, F. Smooth muscle cells (arrows) express Jun (green) in control (**E**) and mutant embryos (**J**) whereas Jun is efficiently deleted in endothelial cells (arrowheads). (**L–O, Q–T**) Higher power images of the area indicated by white boxes in panels K, P. Jun (red) is expressed in SMA-positive smooth muscle cells (green; arrows) in control (**K**) and mutant embryos (**P**). Ao, aorta. Scale bar: 20 µm.

To determine if Jun was required for OFT development and aortic arch artery remodeling, we performed a conditional deletion of *Jun* in endothelium and endothelial-derived endocardium by crossing *Jun* ^flox/flox^ mice with transgenic *Tie2-Cre* mice. To ensure that *Tie2-Cre* was efficiently deleting Jun in endothelial progenitors we performed co-immunostaining for Jun, the endothelial marker, CD31 (Pecam1) and the smooth muscle cell marker, α-smooth muscle actin (SMA). Confocal microscopy of *Jun* ^flox/flox^ and *Tie2-Cre; Jun* ^flox/flox^ embryonic sections revealed that Jun was efficiently deleted in endothelial cells (Fig. 7J) whereas expression in smooth muscle cells was unaffected (Fig. 7J, T).

**Figure 8:**
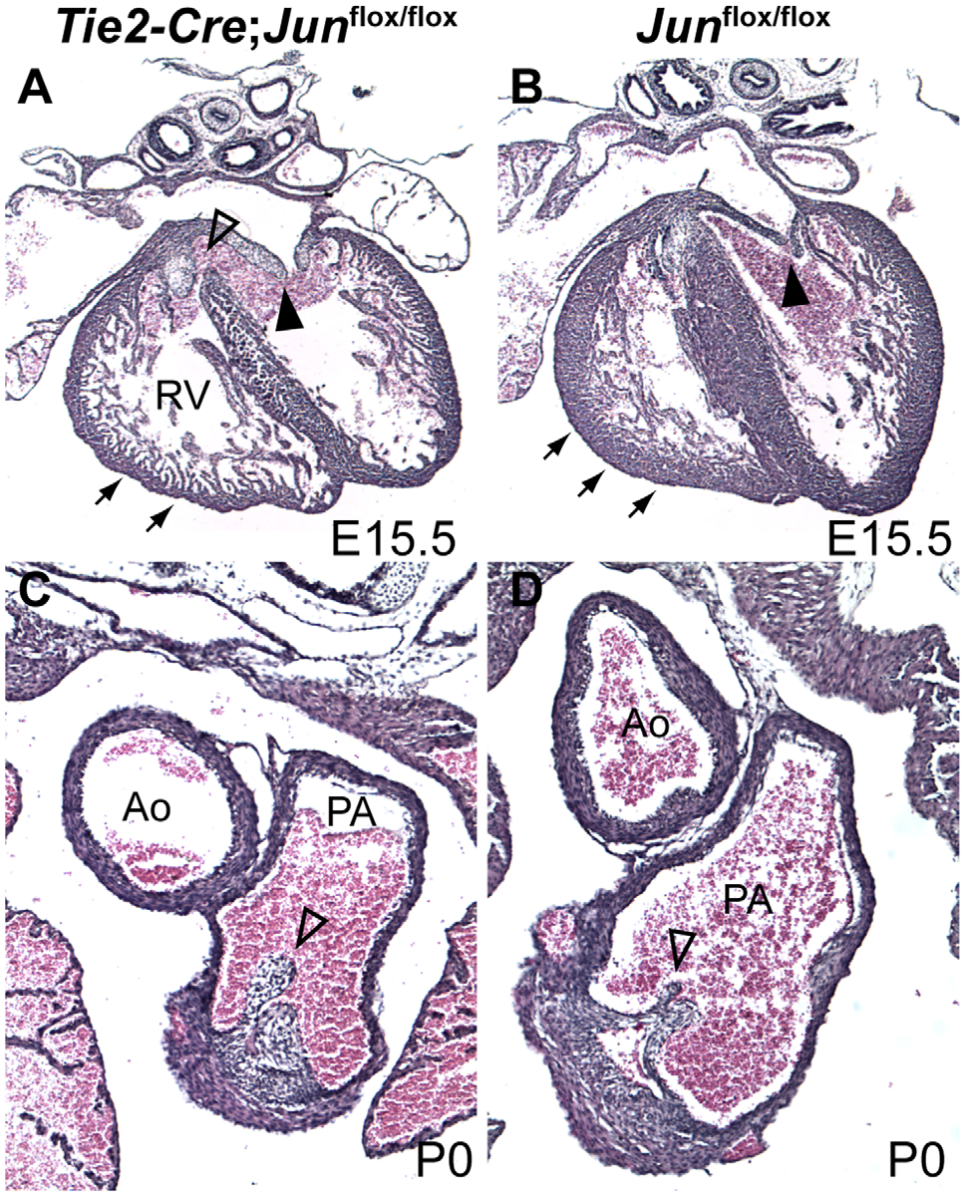
*Tie2*-specific deletion of *Jun* results in cardiovascular defects. Compared to control embryos (**B, D**), the loss of *Jun* in *Tie2*-expressing precursors results in VSD (open arrowhead, **A**), thinned RV (arrows, **A**) and thickened and hyperplastic mitral valve (closed arrowhead, **A**) and pulmonary valve leaflets (open arrowhead, **C**). Ao, aorta; PA, pulmonary artery; RV, right ventricle.

We analyzed embryonic and neonatal *Tie2-Cre; Jun* ^flox/flox^ mutants for a recapitulation of the cardiac phenotype observed in the *Jun* null and *Isl1* conditional *Jun* mutant embryos. We quantified the incidence of embryonic death, aortic arch remodeling defects, and cardiac defects and found that, similar to the *Isl1* ^Cre/+^ *; Jun* ^flox/flox^ embryos, the endothelial-specific knockouts of *Jun* survive until late gestation or even until birth. *Tie2-Cre; Jun* ^flox/flox^ embryos were present at the expected Mendelian ratios (E15.5-P0; Table 1). We examined E15.5 to P0 *Tie2-Cre; Jun* ^flox/flox^ and control embryos for cardiac defects. Upon careful examination, we did not observe any *Tie2-Cre; Jun* ^flox/flox^ conditional mutant embryos (n = 10) with aortic arch artery remodeling defects (Table 2). 43% of conditional mutant embryos had a perimembranous VSD and 14% had double outlet right ventricle, mitral or pulmonary valve hyperplasia (Fig. 8A, C; Table 2). We also noted thinning of the compact myocardium of the right ventricle (Fig. 8A) in 43% (n = 3/7) of *Tie2-Cre; Jun* ^flox/flox^ embryos. This phenotype is similar to that previously described in the global *Jun* null embryos [5]. To determine if these defects were due to a cell non-autonomous effect on NC cell migration, we performed Tfap2a immunostaining of *Tie2-Cre* conditional *Jun* mutant and control embryos. We observed a similar pattern of Tfap2a expression in the developing OFT and pharyngeal arches in E10.5 *Tie2-Cre; Jun* ^flox/−^ embryos compared with controls (Fig. 6G–J) suggesting that defects observed in *Tie2* conditional *Jun* mutant embryos are not attributable to a failure of cardiac NC cell migration. Thus, Jun is not required in endothelial cells and endothelial-derived endocardial cushions for aortic arch artery remodeling or embryonic survival, but is required for the formation of the ventricular septum, compact myocardium and to a lesser extent for formation of the OFT and valves.

## Discussion

Congenital heart disease (CHD) is the most commonly occurring major birth defect in humans with an incidence of 6 per 1000 live births [42]. OFT malformations, including PTA, constitute the largest class of life-threatening CHD. It has been more than a decade since it was recognized that the global loss of Jun in mice uniformly results in PTA [5]. Information regarding Jun expression in cardiac progenitors during critical stages of heart development has been poorly described and thus it has remained unclear in what cell populations Jun may regulate transcription. We show that Jun is expressed in a restricted pattern in several cell populations including the SHF, pharyngeal endoderm, OFT and AV endocardial cushions and post-migratory NC derivatives such as cranial nerve IX and the dorsal root ganglia.

Although *Jun* null embryos die with PTA, we did not observe PTA in conditional *Jun* mutants. There are at least two explanations for this finding. There may be functional redundancy among Jun proteins and/or Jun may be required in other cardiac progenitors not expressing *Isl1*. There is evidence to suggest some degree of functionally redundancy among Jun proteins, such as Jun, Junb and Jund, during heart development. *Jund* ^−/−^ mice have no cardiac defects [43] and *Junb* ^−/−^ null embryos die at E8.5-E10 due to multiple defects in extra-embryonic tissues [44]. Wagner et al. have generated mice in which either *Junb* or *Jund* is knocked-in to the *Jun* locus to test whether Junb or Jund, under the control of the endogenous Jun regulatory elements, can rescue the *Jun* null phenotype [20], [45]. While both of these knock-in mice rescue the mortality at E13 seen in *Jun* ^−/−^ embryos [20], [45], the rescue of the cardiac defects is not as straightforward. *Jun^Junb^* ^/*Junb*^ embryos survive to E18.5 in Mendelian ratios but continue to have PTA (similar to *Jun* ^−/−^ embryos [20]) and VSDs. The same authors then tested whether overexpression of Junb, with *Junb* transgenic mice under the control of human ubiquitin C promoter [46] were able to rescue the *Jun* null phenotype. *Junb*-Tg; *Jun* ^−/−^ embryos are born with no cardiac defects demonstrating redundancy that is dependent on gene dosage [20]. *Jun^Jund^* ^/*Jund*^ embryos survive to E18.5 in Mendelian ratios but also display PTA similar to *Jun* ^−/−^ embryos [45], suggesting that Jund is not functionally redundant for Jun during cardiac development. The rescue of embryonic lethality but not heart defects indicates that different developmental processes have different sensitivities to Jun dosage. It is unclear whether Jund overexpression can rescue the cardiac defects see in *Jun* null embryos. Future studies to test whether decreasing the dosage of Jund or Junb will uncover functional redundancy during heart development may include the analysis of heart defects in *Isl1* ^Cre/+^ *; Jun* ^flox/flox^ *; Junb^+/−^* or *Isl1* ^Cre/+^ *; Jun* ^flox/flox^ *; Jund^+/−^* mutants.

Jun transcriptional activity is dependent on Jun N-terminal kinase (JNK) phosphorylation [47], [48]. This JNK-dependent Jun phosphorylation is dispensable for embryonic development, including for cardiogenesis. Behrens et al. demonstrated this by generating mice in which the *Jun* locus was mutated to prevent JNK phosphorylation (*JunAA*) [49]. *JunAA* homozygous mice, unlike *Jun* ^−/−^ mice, are born in Mendelian ratios and are healthy and fertile, without heart defects, as adults. The notion that JNK-dependent phosphorylation is not essential for heart development is further supported by the observation that *Jnk* mutant mice survive without heart defects [50].

An alternative explanation for the PTA seen in *Jun* null embryos but not in the conditional mutants is that Jun may have roles in multiple cell populations involved in regulating OFT septation. The proximal portion of the OFT cushions (conus) becomes the subpulmonary infundibulum and failure of this structure to form properly results in VSDs. The distal OFT cushions (truncus) gives rise to the semilunar valves and intrapericardial portions of the aorta and pulmonary artery. More distally, the dorsal wall of the aortic sac becomes the aorticopulmonary septum. In humans, defects in this structure result in an aorticopulmonary window [51], [52]. A failure of septation in both the distal OFT and the aortic sac-derived portion of the great arteries results in PTA which usually, but not always [53], is associated with VSDs. The PTA in *Jun* null embryos [5] together with our observation of VSDs in the absence of both PTA and a common truncal valve, supports a model in which “conal” septation, regulated by Jun in *Isl1*-progenitors, is mechanistically separate from both “truncal” and aorticopulmonary septation. Jun may play a role in a non-*Isl1*-expressing domain to regulate “truncal” and aorticopulmonary septation.

Heart development is dependent upon the complex interaction and contribution of several cell types. There are multiple lines of evidence supporting the notion that within the early mesoderm, there is a common cardiovascular progenitor that gives rise to myocardial, smooth muscle and endothelial lineages [54]. Within the splanchnic mesoderm, the SHF, a group of cardiovascular precursors destined to give rise to the right ventricle and OFT, is molecularly marked by the expression of *Tbx1*, a *Mef2c* regulatory module, and by *Isl1* [13], [23], [55], [56], [57]. Data from both murine models and from humans suggest that *ISL1* plays a role in heart development and possibly human CHD. There is a report of a diabetic patient who harbors an *ISL1* mutation [58], but there have been none described in patients with CHD. Despite this, there is evidence to suggest that common *ISL1* single nucleotide polymorphisms (SNPs) are associated with human CHD [59]. It remains to be determined if these SNPs are causative or if they are linked to an associated causative locus. The current study provides data to support a possible role for JUN in ISL1 progenitors and thus adds to the likelihood that this association may be causative. Multipotent Isl1-positive progenitors, when isolated and cultured from either mouse or human embryonic hearts, are capable of differentiating into each of these three lineages [24], [60]. This has also been demonstrated using a potentially overlapping population of Nkx2.5-positive progenitor cells [61]. This *ex vivo* data is consistent with *in vivo* fate-mapping studies, using an *Isl1* inducible Cre, showing the contribution of *Isl1* derivatives to the same three lineages [28]. Recent evidence suggests that some *Isl1*-expressing progenitor cells may also be of a NC lineage (discussed below) [62].

Our analysis of mutants lacking Jun in the endothelial lineage reveals that Jun is required for heart development in some, but not all, *Tie2*-derived endothelial cells and endothelial-derived endocardium. The thinning of the compact myocardium of the right ventricle observed in the *Tie2* conditional *Jun* mutants suggests a model in which there is signaling from the endocardium to regulate the differentiation of primitive myocardial epithelium into compact myocardium. Pathways involved in reciprocal paracrine signaling between the endocardium and myocardium include Neuregulin-1, EphrinB2, Notch, Neurofibromin 1, VEGF, Angiopoietin-1, and Fgf [63], [64], [65], [66], [67]. The role of endocardial Jun in one of these pathways or in a parallel pathway remains to be determined. It is intriguing that there was no compact zone thinning of the left ventricle suggesting that endocardial Jun is not required for the differentiation of *all* primitive myocardial epithelium. Our findings of VSDs in both *Isl1* and *Tie2* conditional *Jun* mutants also support this paracrine model although we cannot conclude from our data whether or not the critical subset of *Tie2*-derived cells is descended from Isl1-positive progenitors. Currently available techniques do not allow us to conditionally delete *Jun* in *Tie2/Isl1*-positive progenitors *in vivo* while leaving *Jun* unaltered in *Tie2*-positive *Isl1*-negative progenitors. Thus, it remains to be tested if Jun is dispensable in the *Isl1*-negative endothelial lineage. Alternatively, the VSDs we observed may be a common phenotype resulting from independent requirement for Jun in both *Isl1*-negative endothelial/endocardial cells and *Tie2*-negative *Isl1* progenitors.

Our finding of aortic arch artery patterning defects in *Isl1*-specific Jun deleted embryos raises the possibility of a cell autonomous effect of Jun in an *Isl1*-derived smooth muscle lineage or alternatively a cell non-autonomous effect on another cell population such as NC. There are other examples of genetic alterations in the SHF affecting a tissue-tissue interaction with NC cells. We have shown that deletion of Notch in the SHF using *Isl1* ^Cre^ or *Mef2c*-AHF-Cre results in severe NC-related cardiac defects including PTA and IAA [36], defects identical to those seen in our *Isl1* ^Cre/+^; *Jun* ^flox/flox^ embryos and in *Jun* null embryos [5]. Further tissue-restricted deletion studies are required to determine the relative requirement for Jun specifically in the smooth muscle or myocardial lineages.

Our expression analysis indicates that Jun is expressed in post-migratory NC derivatives. NC cells are a specialized subset of neuroepithelial cells in the dorsal neural tube that migrate ventrally and contribute to a diverse array of tissues. Raivich et al. have previously reported that Jun is expressed in neuroepithelial cells [68] yet deletion of Jun in neuroepithelial cells with *Nestin*-Cre did not result in congenital heart defects [30], [69]. It is unclear if *Nestin*-Cre is expressed in a cardiac NC subset of neuroepithelial cells. Cardiac NC cells, originating between the mid-otic placode and the third somite, invade the pharyngeal arches and encompass the aortic arch arteries around E10. By E10.5 they populate the cardiac OFT as two columns of cells subsequently forming a portion of the mesenchymal tissue in the OFT endocardial cushions. Notably, these cushions are abnormal in *Jun* null embryos [5]. Ultimately, descendants of this subset of NC cells contribute to the aorticopulmonary septum, dividing the truncus arteriosus into the aorta and pulmonary artery [10], [70], [71]. Although there is evidence that the complete loss of *Jun* does not globally affect the fate of NC cells, by E12.5 there are fewer connexin43-labeled cardiac NC cells populating the right ventricular OFT of *Jun* null embryos [5]. In contrast, our findings suggest that the defects observed in *Isl1* and *Tie2* conditional *Jun* mutant embryos are not attributable to a cell non-autonomous defect of cardiac NC migration. In the global *Jun* null embryo, it is unknown whether the fewer connexin43-labeled cells is due to a secondary cell non-autonomous mechanism affecting NC as the result of a loss of Jun function in another tissue such as SHF or pharyngeal endoderm. This is further complicated by the recent observation that some *Isl1*-expressing progenitor cells may be of a NC lineage [62]. This novel and surprising data raises the possibility that the defects observed in *Isl1*-conditional *Jun* mutants may reflect a cell autonomous defect of NC. The effect of conditional deletion of *Jun* in *Isl1*-expressing progenitors on NC cell differentiation was not determined in the current study but is an important area for future investigation. Tissue-restricted deletion studies using *Wnt1*-Cre or *Pax3* ^Cre/+^ mice [72], [73] are required to determine the requirement for Jun specifically in NC derivatives.

Jun, as part of the AP-1 transcription factor complex, is a positive regulator of cell proliferation [7], [8], [32] and positively regulates cell cycle progression through p19, p53, cyclin D1 and cyclin A pathways [6], [8], [29], [74], [75], [76], [77]. It regulates the differentiation of varied cell populations such as hematopoietic cells and keratinocytes [6], [9] and has also been shown to regulate apoptosis in such cells as fibroblasts, hepatocytes and neurons [6], [8], [78], [79]. Although these pleiotropic cellular functions are likely to be important for all progenitors, our observation that *Isl1-* and *Tie2*-specific *Jun* knockouts survive embryogenesis in Mendelian ratios indicates that Jun is dispensable in these progenitors for overall embryonic survival. Though Jun is not required for formation or maintenance of the blood vessels (at least after the time of *Tie2*-Cre-mediated recombination), it is not completely dispensable in endothelial precursors as they display a low incidence of cardiac defects. Out data do not exclude the possibility of subtle defects in endothelial cell function in these *Jun* mutant embryos.

The molecular mechanisms by which Jun functions during heart development, in *Isl1*-expressing cells and other cells remains to be determined. In the embryonic human heart, ISL1 is strongly expressed in highly proliferative SHF-derived cell populations in the arterial and venous poles [80]. We observed a statistically insignificant trend toward a decrease in proliferating cells and an increase in apoptosis in the OFT of mutant embryos. These individual differences were modest when compared with controls, but in combination these two alterations may contribute to defects in OFT septation or complete formation of the ventricular septum. There are several transcriptional networks and signaling pathways known to regulate proliferation in the SHF. These include *Nkx2.5*, Wnt/ß-catenin, Notch, Fgf, Shh, *Isl1*, and *Tbx1* signaling pathways (reviewed in [13]). *TBX1* is a molecular marker of the SHF and accruing evidence points to a causative role for this transcription factor in the pathogenesis of DiGeorge syndrome (DGS). It has been proposed that the loss of *TBX1* in patients with DGS results in defective proliferation in the SHF [81], [82], although little mechanistic data have yet been published to support this notion. Cardiac defects observed in patients with DGS and in *Tbx1* mutant mice are strikingly similar to those seen in *Jun* mutant mouse embryos [83], [84], [85] and to mice in which NC has been disrupted [10]. Despite the similarity with NC mutants and reports of a disruption in the distribution of NC-derived cells in *Tbx1* nulls, *Tbx1* is not expressed in NC [86]. One hypothesis is that Jun and Tbx1 could be acting in concert to regulate the proliferation of SHF progenitors and subsequently affect interactions with other tissues such as NC. Future studies are required to determine if Jun may function in a Tbx1-dependent pathway and to further elucidate cell autonomous and cell non-autonomous mechanisms of Jun function during heart development.

## Materials and Methods

### Ethics Statement

Animal studies were conducted in accordance with the National Institutes of Health National Research Council Guide for the Care and Use of Laboratory Animals and were approved by the Children’s Hospital of Philadelphia Research Institute Institutional Animal Care and Use Committee (No. 2008-6-840; Department of Health and Human Services Animal Welfare Assurance #A3442-01).

### X-Gal Staining

Whole mount embryos were stained for ß-galactosidase activity using previously described methods [87].

### Confocal Microscopy

Confocal images were acquired with the Zeiss LSM 510/NLO META confocal microscope using 20x, 0.8 NA Plan-Apochromat air immersion and 63x, 1.4 NA Plan-Apochromat oil immersion objectives.

### Mutant Mice and Genotyping

All mouse strains used are previously described and listed here: *Jun* ^flox/flox^ is *Jun^tm4Wag^* [7], *Isl1* ^Cre/+^ is *Isl1^tm1(cre)Sev^* [25], *Tie2*-Cre is Tg(Tek-cre)1Ywa [41], R26R is *Gt(ROSA)26Sor^tm1Sor^* [40], mT/mG is *Gt(ROSA)26Sor^tm4(ACTB−tdTomato,−EGFP)Luo^* [34], and *Jun* ^+/−^ is *Jun^tm1Pa^* [4]. The last four lines were obtained from the Jackson Laboratory (Bar Harbor, ME). Mouse genotyping was performed using real-time quantitative polymerase chain reaction (qPCR) techniques. Genomic DNA was isolated with the Extract-N-Amp Tissue PCR kit (Sigma-Aldrich, St. Louis, MO) according to the manufacturer’s recommended protocol. PrimeTime qPCR assays (Integrated DNA Technologies, Coralville, IA) consisting of the primer and probe sequences listed in Table S1 were used. The mouse GAPD TaqMan Gene Expression Assay (Life Technologies, Carlsbad, CA) was used as the endogenous control.

### Immunohistochemistry

Immunofluorescence (IF) and horseradish peroxidase (HRP) immunostaining were performed as described previously [88] using rabbit monoclonal anti-Jun 60A8 (1∶100 IF; Cell Signaling Technology, Danvers, MA), rat monoclonal anti-CD31 MEC13.3 (Pecam1; 1∶100; BD Pharmingen, Franklin Lakes, NJ), mouse monoclonal anti-Isl-1 39.4D5 (1∶25 IF; Developmental Studies Hybridoma Bank, Iowa City, IA), rabbit polyclonal anti-GFP (1∶200 IF, Life Technologies), mouse monoclonal anti-SMA 1A4 (1∶200 IF, Life Technologies), rabbit polyclonal phospho-histone H3 Ser10 (1∶200 IF, Cell Signaling Technology), mouse monoclonal AP-2 alpha (Tfap2a) 3B5 (1∶4 IF, Developmental Studies Hybridoma Bank) and mouse monoclonal NFATc1 7A6 (1∶25 IF, Developmental Studies Hybridoma Bank). TUNEL assays were performed using the In Situ Cell Death Detection Kit (Roche, Indianapolis, IN) according to the manufacturer’s recommended protocol. Images were quantified by using the Image-based Tool for Counting Nuclei (ITCN) plug-in (http://goo.gl/fPQ6B) for ImageJ (NIH, Bethesda, MD). The mean and SEM were calculated based on three independent sections per embryo.

### Whole Mount in situ Hybridization

Whole mount *in situ* hybridization was performed as described [89] with modifications. Digoxigenin-UTP-labeled RNA probes were generated from plasmid template containing mouse *Jun* (IMAGE clone 3493248; GenBank accession number BC002081) by *Xma*I restriction digestion and transcription with T7 RNA polymerase. Embryos were fixed in 4% PFA overnight, then dehydrated in a graded series of methanol in PBST. The embryos were serially rehydrated to PBST. Embryos were permeabilized in RIPA buffer thrice for 5 minutes at room temperature. Embryos were refixed in 4% PFA/0.2% glutaraldehyde in PBS for 20 minutes, washed and hybridized overnight at 70°C in hybridization solution containing 1 µg probe/mL. Embryos were washed and hybridized with alkaline phosphatase conjugated anti-digoxigenin antibody (Roche) overnight at 4°C, washed for 24 hours and developed in BM purple (Roche).

## Supporting Information

Figure S1 **Jun is broadly expressed in the late gestation mouse heart.** (**A, E**) Transverse sections of an E15.5 wild-type mouse heart showing nuclear Jun immunostaining (green) in the myocardium and valves. (**B–D**) Higher power images of the areas shown in the white boxes in panel A showing Jun expression in the myocardium and atrioventricular valves. (**F**) Higher power image of the area shown in the white box in panel E showing Jun expression in the right ventricular outflow tract myocardium and pulmonary valve. Sections were co-stained with DAPI to illustrate nuclei. Ao, aorta; IVS, interventricular septum; LA, left atrium; LV, left ventricle; MV, mitral valve; PA, pulmonary artery; PV, pulmonary valve; RA, right atrium; RV, right ventricle; TV, tricuspid valve.
(TIF)

Table S1 **qPCR Genotyping Assays.** Primer and probe sequences used for mouse genotyping. Mouse GAPD was used as the endogenous control.
(DOC)
