# Supplementary figures and images for: Jun Is Required in Isl1-Expressing Progenitor Cells for Cardiovascular Development
Source: PLoS One. 2013 Feb 21;8(2):e57032. doi: 10.1371/journal.pone.0057032 (PMC3578783; doi:10.1371/journal.pone.0057032)

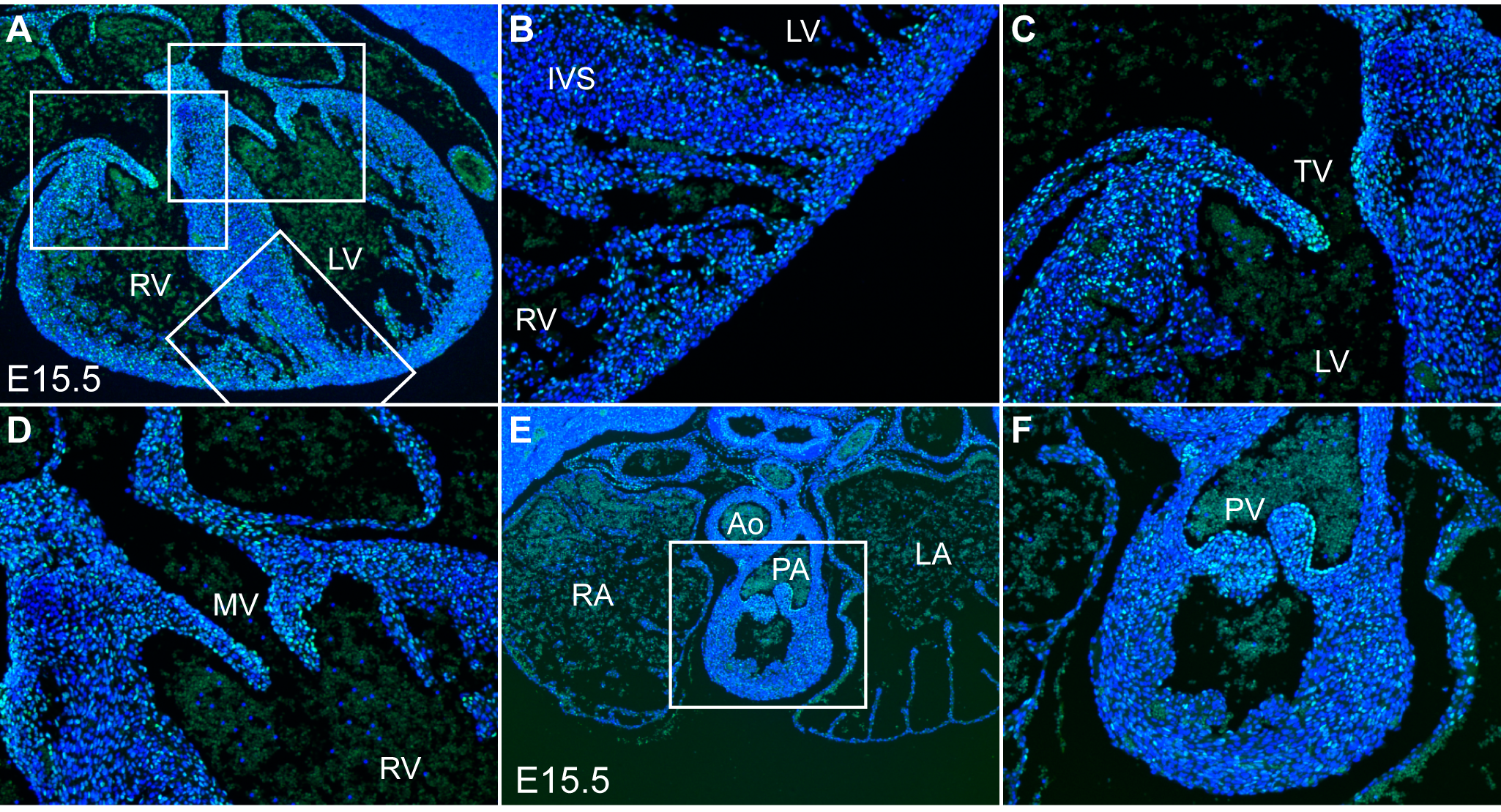

Supplement: Figure S1 — Jun is broadly expressed in the late gestation mouse heart. (A, E) Transverse sections of an E15.5 wild-type mouse heart showing nuclear Jun immunostaining (green) in the myocardium and valves. (B–D) Higher power images of the areas shown in the white boxes in panel A showing Jun expression in the myocardium and atrioventricular valves. (F) Higher power image of the area shown in the white box in panel E showing Jun expression in the right ventricular outflow tract myocardium and pulmonary valve. Sections were co-stained with DAPI to illustrate nuclei. Ao, aorta; IVS, interventricular septum; LA, left atrium; LV, left ventricle; MV, mitral valve; PA, pulmonary artery; PV, pulmonary valve; RA, right atrium; RV, right ventricle; TV, tricuspid valve. (TIF) [file pone.0057032.s001.tif]
