# Supplementary material for: Jun Is Required in Isl1-Expressing Progenitor Cells for Cardiovascular Development
Source: PLoS One. 2013 Feb 21;8(2):e57032. doi: 10.1371/journal.pone.0057032 (PMC3578783; doi:10.1371/journal.pone.0057032)
Supplement: Table S1 — qPCR Genotyping Assays. Primer and probe sequences used for mouse genotyping. Mouse GAPD was used as the endogenous control. (DOC) [file pone.0057032.s002.doc]

Table S1: qPCR Genotyping Assays

| Mouse Strain(s) | Assay Name | Primer/Probe sequence |
| --- | --- | --- |
| *Isl1*^Cre/+^, *Tie2-Cre* | Cre | Primer 1: 5’-CCAGCAACATTTGGGCCAGCTAAA-3’ |
|  |  | Primer 2: 5’-GCCGCATAACCAGTGAAACAGCAT-3’ |
|  |  | Probe: 5’-TGCCACGACCAAGTGACAGCA-3’ |
| *Jun*^+/–^ | Jun-WT | Primer 1: 5’-GACAGTTGTCGGAGGCC-3’ |
|  |  | Primer 2: 5’-GACCAGTCCCAGCAACAG-3’ |
|  |  | Probe: 5’-ACGGAATTCTCGGTGGAAGCCC-3’ |
|  | Jun-mutant | Primer 1: 5’-TGGGTCGTTTGTTCGGATC-3’ |
|  |  | Primer 2: 5’-GACCAGTCCCAGCAACAG-3’ |
|  |  | Probe: 5’-ACCAGTCGTCACGGAATGCCAA-3’ |
| *Jun*^flox/flox^ | Jun-WT | Same as above |
|  | Jun-CN-mutant | Primer 1: 5’-GGCTTCCACCGAGAATTCAAG-3’ |
|  |  | Primer 2: 5’-GACCAGTCCCAGCAACAG-3’ |
|  |  | Probe: 5’-TGCTGACCAGTCGTCACGGAATTATAAC-3’ |
| R26R | LacZ | Primer 1: 5’-TTATCAGCCGGAAAACCTACC-3’ |
|  |  | Primer 2: 5’-CTCGCCACTTCAACATCAAC-3’ |
|  |  | Probe: 5’-TCGCCATTTGACCACTACCATCAATCC-3’ |
